# Transport decoupling in glasses begins in the high-temperature activation landscape

**DOI:** 10.1093/nsr/nwag436

**Published:** 2026-07-16

**Authors:** Alessio Zaccone

**Affiliations:** Department of Physics ‘Aldo Pontremoli’, University of Milan, Italy

The breakdown of the Stokes–Einstein relation is among the clearest dynamical signatures of approaching vitrification. In a normal liquid, diffusion and structural relaxation are locked together and *D* ∼ *τ*^−1^, where *D* is the diffusion coefficient and *τ* is the Maxwell relaxation time. In a deeply supercooled liquid, in contrast, diffusion remains anomalously efficient compared with viscosity or the structural relaxation time, and the relation is commonly replaced by a fractional form, *D* ∼ *τ*^−^*^ξ^*, where *ξ* is a fractional exponent. The standard explanation invokes dynamic heterogeneity: spatially intermittent regions of mobility emerge, with fast regions controlling diffusion and slow regions controlling relaxation.

**Figure 1.**
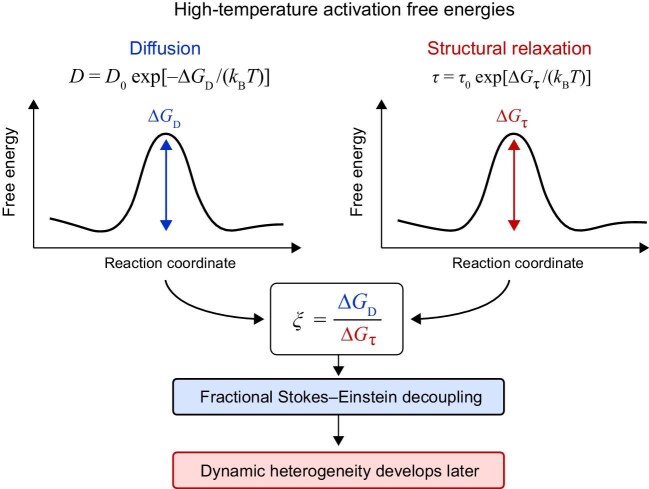
Effective activation free energies for diffusion (Δ*G*_D_) and structural relaxation (Δ*G*_τ_), extracted from high-temperature Arrhenius dynamics, determine the fractional Stokes–Einstein exponent, *ξ* = Δ*G*_D_/Δ*G_τ_*. The conceptual advance of Yuan *et al.* is that transport decoupling is already encoded in these activation free energies in the high-temperature normal liquid, while dynamic heterogeneity emerges only later upon supercooling.

Yuan *et al.* [1] now put this familiar causal interpretation under pressure. By analysing several model glass-forming liquids, they show that the fractional decoupling exponent is predicted by a ratio of activation free energies determined in the high-temperature Arrhenius regime (Fig. 1). This regime precedes the strong growth of dynamic heterogeneity. The implication is therefore not merely quantitative, but conceptual: the fingerprints of Stokes–Einstein breakdown appear to be written into the normal liquid before the heterogeneous dynamics, normally blamed for the breakdown, have fully developed.

This conclusion strengthens a broader view that glassy anomalies are controlled by activation physics already present above the onset of strongly cooperative motion. Energy-landscape descriptions, elastic models, random first-order transition theory, and the string model have all emphasized activated rearrangements, although from different microscopic perspectives [2–6]. The advance of Yuan *et al.* is to turn the fractional Stokes–Einstein exponent from a phenomenological descriptor into a quantity predictable from high-temperature activation free energies.

The result also sharpens the next theoretical question. If the exponent is encoded in activation barriers, what microscopic variables determine those barriers? Local cage rigidity [4], nonaffine elasticity [7], stress redistribution, and correlated rearrangements [8] are natural candidates. Atomistic and elastic approaches based on local relaxation events have shown how interactions between rearrangements can generate non-Debye relaxation, evolving barriers and correlated dynamics [8,9]. Related work has further connected liquid fragility and viscoelasticity to the steepness of the local energy landscape through microscopic elasticity and thermal expansion, providing a route from microscopic interactions to macroscopic glassy dynamics [10]. In this light, the new work complements microscopic barrier theories: it demonstrates that the early, high-temperature values of the relevant barriers already have predictive power for the anomalous transport observed much later upon cooling.

The practical consequences may be significant. Transport decoupling influences crystallization, ionic conduction, polymer processing, metallic-glass stability, and the ageing of amorphous materials. Predicting decoupling from high-temperature data would make an intrinsically difficult low-temperature property accessible in a regime where experiments and simulations are more tractable.

By reversing the usual causal arrow, Yuan *et al.* shift attention from dynamic heterogeneity itself to the activation landscape from which heterogeneity and decoupling jointly emerge. Their study suggests that anomalous glassy transport is not born abruptly near the glass transition; it is foreshadowed by the high-temperature activation free energies of the normal liquid.
